# A Novel Fault Location Method for a Cross-Bonded HV Cable System Based on Sheath Current Monitoring †

**DOI:** 10.3390/s18103356

**Published:** 2018-10-08

**Authors:** Mingzhen Li, Chengke Zhou, Wenjun Zhou, Chunlin Wang, Leiming Yao, Mengting Su, Xiaojun Huang

**Affiliations:** 1 School of Electrical Engineering, Wuhan University, No. 299, Bayi Road, Wuchang District, Wuhan 430072, China; wjzhou@whu.edu.cn; 2 School of Engineering and Built Environment, Glasgow Caledonian University, Cowcaddens Road, Glasgow G40BA, UK; 3 State Grid Jiangsu Electric Power Company, State Grid Corporation of China, No. 555, Laodong Road, Gusu District, Suzhou 215000, China; whjetli@gmail.com (C.W.); kids7762@sina.com (L.Y.); smt.and.gd@gmail.com (M.S.); mxc60@163.com (X.H.)

**Keywords:** circuit faults, electromagnetic time reversal, fault currents, fault location, power cables

## Abstract

In order to improve the practice in the operation and maintenance of high voltage (HV) cables, this paper proposes a fault location method based on the monitoring of cable sheath currents for use in cross-bonded HV cable systems. This method first analyzes the power–frequency component of the sheath current, which can be acquired at cable terminals and cable link boxes, using a Fast Fourier Transform (FFT). The cable segment where a fault occurs can be localized by the phase difference between the sheath currents at the two ends of the cable segment, because current would flow in the opposite direction towards the two ends of the cable segment with fault. Conversely, in other healthy cable segments of the same circuit, sheath currents would flow in the same direction. The exact fault position can then be located via electromagnetic time reversal (EMTR) analysis of the fault transients of the sheath current. The sheath currents have been simulated and analyzed by assuming a single-phase short-circuit fault to occur in every cable segment of a selected cross-bonded high voltage cable circuit. The sheath current monitoring system has been implemented in a 110 kV cable circuit in China. Results indicate that the proposed method is feasible and effective in location of HV cable short circuit faults.

## 1. Introduction

In recent years, power cables have been widely used in urban transmission and distribution systems due to their aesthetics and high reliability [1,2,3]. With the rapid growth of power cable usage, the number of cable short circuit faults has increased [3,4]. Efforts are needed to localize short circuit faults in power cable systems accurately and in a timely manner.

Currently, two popular methods have been adopted for the fault location of HV cable systems. One is the relay compensation method using data collected from distance protection devices [5,6,7], and the other is the traveling wave method [8,9,10,11]. The theory of distance protection is based on parameter identification, as the system parameter would change when a fault occurs [6]. However, in situations where a power cable circuit may contain a few major sections and/or overhead lines, it is difficult to determine accurately the relationship of impedance versus distance [7], since the measured impedance may not be linearly proportional to the fault distance. The theory of the traveling wave method is based on an analysis of the propagation time of the transient wave that is associated with fault current [8,9,10,11]. However, problems lie with noise elimination and the accurate identification of the wave head.

Recently, there have been reports of two kinds of improvements to the impedance and traveling wave methods. One is a fault location method for use in double circuit, medium power distribution networks [12]. The method takes into account the mutual inductive effect of double circuit lines, and it is applicable to fault location in scenarios of double circuit lines. The other is the electromagnetic time reversal (EMTR) method [13,14,15,16]. The theory behind the method is based on the solution of the wave equation. The EMTR fault location method was firstly applied to a lossless line and proven to be theoretically effective [13]. Then, the influence of the losses was assessed when the method was applied to real world power systems [14,15,16]. However, the two methods both require detailed system topology, including line and cable parameters that may not be readily available in practice.

The present authors previously proposed a fault localization method for fault segment location in conference contributions [17,18]. The present paper expands on their previously published work, and makes modifications of the expressions for use in fault location in a cross-bonded HV cable system. There are two main steps in the proposed fault location method: the first is to locate the fault segment based on the power–frequency phase difference of the sheath currents at the two ends of each cable segment, and the second is to locate the exact fault position based on a modified EMTR (electromagnetic time reversal) method. The first step of the proposed method is unique in that the exact cable segment with a fault can be identified with confidence, due to the distributed sheath current monitoring. This is important for cable maintenance engineers, as the result allows them to carry out further test and repair/replacement of the faulty cable segment or accessory in a timelier manner. Past inaccuracies in fault location have led to testing of wrong cable segments and have resulted in lengthy delays in cable failure restoration in the past.

The paper firstly introduces an online condition monitoring system that has been designed to monitor the sheath currents of a HV cable system. Then, the sheath current signals under various scenarios when short circuit faults occur in different segments are analyzed using PSCAD (Power System Computer Aided Design), and a set of criteria based on it is proposed for fault segment location. The EMTR method is then modified for the purpose of exact fault point location using components of the sheath current transients at either end of the fault segment. Finally it presents the practical implementation of the proposed system in a 110 kV cable system, and practical data collected from the system, with which the effectiveness of the fault location method is evaluated.

## 2. Cross-Bonded Cable Sheath Currents and Proposed Online Monitoring System

Cable sheath currents depend on the asymmetry of the three-phase load currents, the laying methods, the length of cables in each of the minor sections, the number of major sections and external electromagnetic environment [19,20,21,22]. HV cable sheath currents contain induced current and leakage current. The induced current is the main part of sheath current.

**Figure 1:**
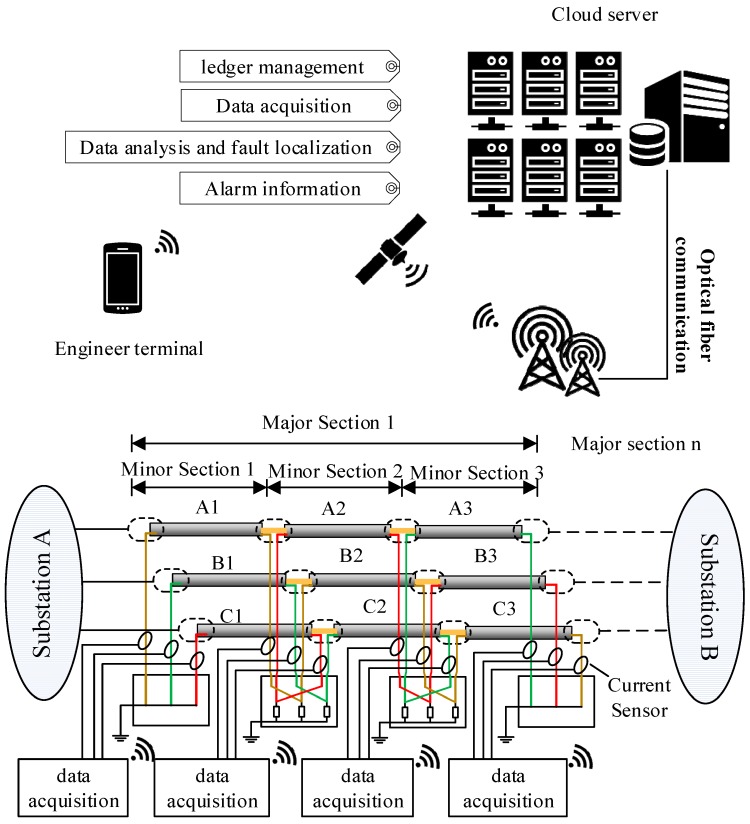
The configuration of a cross-bonded HV cable and its online sheath currents monitoring system.

Cross-bonding is one of the main features of a HV cable system. To reduce the unbalanced three-phase load effects on sheath currents, long HV cable circuits (>1.2 km) usually have their metal sheath or the conductors transposed every 400~500 m, as shown in Figure 1. It is to be noted that a whole cross-bonding section (referred to as the “major section” in [19]) consists of three adjacent cable sections or cable segments (referred to as “minor sections” in [19]), of which the metal sheaths are cross-bonded. The metal sheath is directly connected to the ground through the grounding boxes G1 and G2 at both ends of a major section. At the joints J1 and J2, the sheaths are connected to ground via overvoltage limiters.

Figure 1 also shows the same HV cross-bonded cable system where the proposed system is being implemented. In order to measure the sheath currents, current sensors are installed at grounding boxes G1 and G2, and cross-bonding link boxes J1 and J2. The sheath currents detected at G1 are denoted as *I*_1a_, *I*_1b_, *I*_1c_. Likewise, the sheath currents detected at J1 are *I*_2a_, *I*_2b_, *I*_2c_. The sheath currents detected at J2 are *I*_3a_, *I*_3b_, *I*_3c_. The sheath currents detected at G2 are *I*_4a_, *I*_4b_, *I*_4c_.

**Figure 2:**
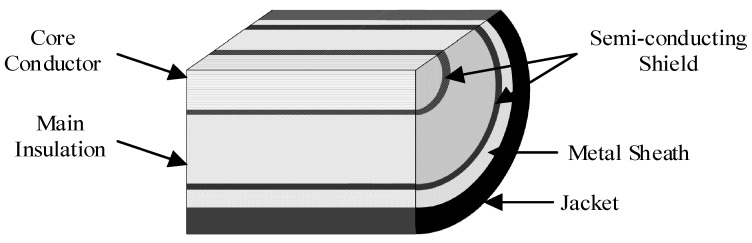
A typical structure of a single core HV cable.

When a breakdown occurs between the main conductor and the metal sheath in a cable segment, the resultant fault current will flow into the metal sheath along both directions to the ground. The sheath current in the loop where the fault happens will rise to the level of fault current. Meanwhile, because of the electromagnetic coupling effect, cables in the other phases will also induce large currents. This paper uses the fault current in the metal sheath, between the instant of fault occurring and the moment the fault is cleared, to locate the fault. As shown in Figure 2, the system contains four parts, namely, the data acquisition module, the communication module, the location analysis software installed in a cloud server, and the interface for the final users, e.g., the cable maintenance engineers. The data acquisition module has two sets of current sensors: one for power-frequency sampling, the other for high frequency (fault transients) sampling. The data acquisition module is capable of being woken up and commencing data upload within 5 ms, whilst it takes around 70 ms to 100 ms for the protection system to clear any fault. As the data acquisition module is designed to have a caching mechanism, the data is stored in the cache first. It will only be uploaded when the trigger threshold is reached. Therefore, the entire system is not very resource demanding, and it is not very expensive. The communication module of the data acquisition system can transmit the recorded data to a designated cloud server, where the location analysis software carries out data analysis before sending the location results to the maintenance engineers. The recoded data can also be downloaded from the server for further analysis.

## 3. The Cable Model for Fault Location

An IEEE standard [19] and a CIGRE (International Council on Large Electric Systems) technical brochure [23] provided the procedures for sheath voltage and current calculations under steady state. They also indicated how cable sheath currents can be calculated under fault situations. The models of cable circuit representations and sheath current calculations in the present paper were in line with the standards. However instead of using EMTP/ATP, the authors used PSCAD for numerical simulations.

**Figure 3:**
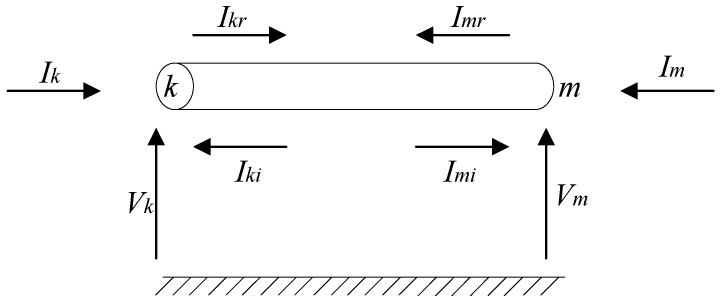
Equivalent two-port network of the cable.

A cable can be modeled as a two-port network, as shown in Figure 3. The equivalent network could be defined by two transfer function matrixes [22,23]: the transfer function matrix *H* and the admittance matrix *Yc*, as presented in Equation (1).

(1) $$\begin{array}{l} Y_{c}\cdot V_{k}-I_{k}=2\cdot H^{T}\cdot I_{mr}=2\cdot I_{ki}\\ Y_{c}\cdot V_{m}-I_{m}=2\cdot H^{T}\cdot I_{ki}=2\cdot I_{mi} \end{array}\}$$

Here, *H* and *Y_c_* could be represented by Equations (2) and (3):

(2) $$H=e^{-\sqrt{Z\cdot Y}\cdot l}$$

(3) $$Y_{C}=Z^{-1}\sqrt{ZY}$$

*Z* and *Y* are the series impedance and shunt admittance per unit length; *l* stands for the length of the cable; *V_k_* and *V_m_* are the voltage vectors at nodes *k* and *m*; *I_k_* and *I_m_* are the current vectors at node k and m; *I_kr_* and *I_mr_* are the reflective current vectors at nodes *k* and *m* respectively.

Equations (1)~(3) mean that the transmission characteristics could be expressed by *H* and *Y_c_*. *H* and *Y*c could be determined by *Z* and *Y*. The basic formulae describing a transmission line system are given in Equations (4) and (5) [23,24]:

(4) $$\frac{dV}{dx}=-Z\cdot I$$

(5) $$\frac{dI}{dx}=-Y\cdot V$$

## 4. Analysis of Fault Current and Criteria for Fault Location

### 4.1. Simulation and Analysis of Cable Sheath Currents

Simulation has been carried out for a 110 kV HV cable circuit using PSCAD. All cable segments have a conductor cross-section of 800 mm^2^. The parameters of the cross-sectional structure are shown in Figure 2 and Table 1.

**Figure 4:**
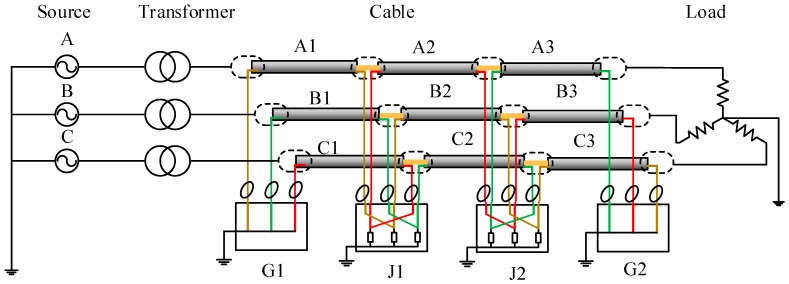
Schematic diagram of the cable system.

The power network in simulation, as shown in Figure 4, is a simple power system containing a power source, a transformer, and a major section of the cross-bonded cables and loads, where three phase cable system with three minor sections (nine cable segments for the three phases) are installed in a flat horizontal formation. Each minor section is 500 m, hence the total cable circuit length is 1500 m. The grounding resistance in each of the sheath loops is 0.1 Ω.

Assuming the three-phase voltages of the 110 kV power source are *U_a_* = 63.51∠0° kV, *U_b_* = 63.51∠−120° kV, *U_c_* = 63.51∠120°, and that the balanced load is 40 MW. Then, the three phase currents from simulation results are: *I_a_* = 209.97∠−1.3° A, *I_b_* = 210.00∠−121.3° A, *I_c_* = 210.05∠118.7° A, respectively.

Considering the axial distribution and radial structure, the cable circuit is expressed as a distributed parameter model. Therefore the voltage and current in any location of the cable model is not exactly the same. The magnitude of the sheath current under healthy conditions in each of the sheath loops is only a few amperes.

**Figure 5:**
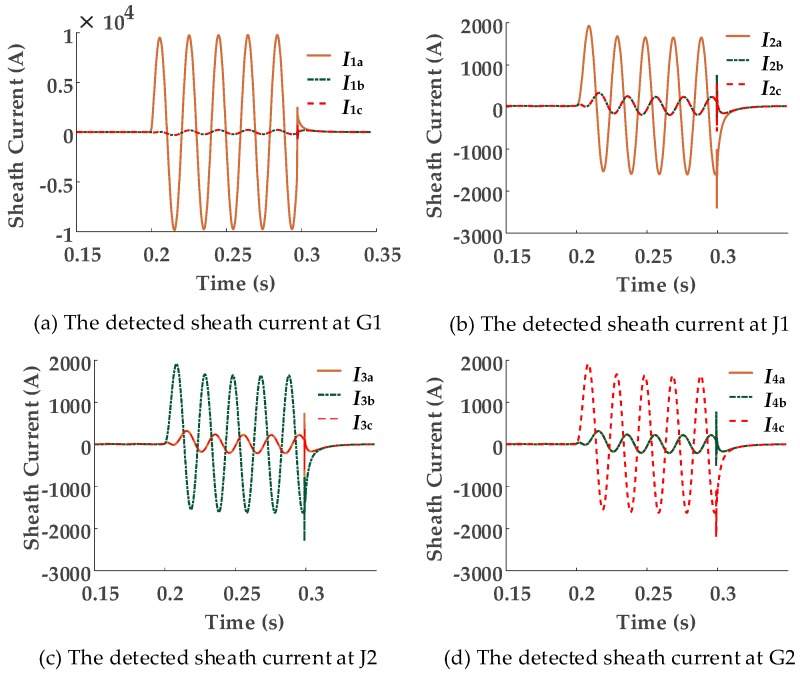
The simulation results of sheath currents during a short circuit fault. (**a**) The detected sheath current at G1 (**b**) The detected sheath current at J1 (**c**) The detected sheath current at J2 (**d**) The detected sheath current at G2.

Assuming a single-phase short circuit fault occurs in cable segment A1. The fault duration is 0.1 s. The fault position is 300 m from the left cable terminal of Figure 1. The simulation results of each sheath current at each detection point are presented in Figure 5.

Figure 5 shows that, when a breakdown happens in cable segment A1 between the conductor and the sheath, the resultant fault current in section 1, phase A, splits in two directions in the sheath when flowing to ground. Part of the fault current flows from the breakdown point to the grounding point in G1, whilst, the other part of the fault current flows from the breakdown point to the grounding point in G2, causing a high level of sheath currents *I*_1*a*_, *I*_2*a*_, *I*_3*b*_, and *I*_4*c*_ to be detected at G1, J1, J2, and G2 respectively. Meanwhile, the sheath currents in phases B and C also increase, owing to mutual coupling (This explains why currents in the two healthy phases are almost identical in Figure 5). Because of the cross-bonded connection, the fault currents detected at other detection points flow along the transposed sheaths before flowing to ground, as shown in Figure 5b–d.

In this section of the paper, only the fundamental signal under 50 Hz when establishing the criteria for fault segment location is analyzed. The magnitudes of the 50 Hz currents at each of current sensors within different fault segments are shown in Table 2.

Table 2 indicates that the magnitudes of fundamental current at each of the sensor positions vary with the change in segment where a fault occurs. Due to the cross-bonded connection, it is not always possible to determine the faulted segments based on the sheath current magnitudes. When a fault occurs in A1, B1, C1, A3, B3, or C3, it is relatively easy to recognize the fault segment. However, when a fault occurs in segment A2, B2, or C2, especially when the fault point is near the middle of the cable segments, it is impossible to differentiate the fault segment based only on an analysis of the sheath current magnitudes.

### 4.2. Criteria for Fault Segment Location

Fault currents flow in both directions along the metal sheath to ground, for the cable segment where a fault occurs. Thus, the phase difference between the currents flowing towards the two ends of the cable segment is nearly 180°. Generally, the length of each cable segment is no more than 500 m. In practice, each of the three cable segments may have a different length. However, the phase shift caused due to unequal section lengths between the detected currents at the two ends is not noticeable. Let *B*(I) be the phase angle of the fundamental frequency current I. *P*(segment) is the phase difference between the sheath currents at either side of a cable segment (segment ∈ {“A1” “B1” “C1” “A2” “B2” “C2” “A3” “B3” “C3”}). Consequently, the calculation of the phase difference is presented in Equation (6). The results are shown in Table 3.

(6) $$\begin{array}{l} P\left(A1\right)=B\left(I_{2a}\right)\;-\;B\left(I_{1a}\right)\\ P\left(B1\right)=B\left(I_{2b}\right)\;-\;B\left(I_{1b}\right)\\ P\left(C1\right)=B\left(I_{2c}\right)\;-\;B\left(I_{1c}\right)\\ P\left(A2\right)=B\left(I_{3a}\right)\;-\;B\left(I_{2c}\right)\\ P\left(B2\right)=B\left(I_{3b}\right)\;-\;B\left(I_{2a}\right)\\ P\left(C2\right)=B\left(I_{3c}\right)\;-\;B\left(I_{2b}\right)\\ P\left(A3\right)=B\left(I_{4a}\right)\;-\;B\left(I_{3c}\right)\\ P\left(B3\right)=B\left(I_{4b}\right)\;-\;B\left(I_{3a}\right)\\ P\left(C3\right)=B\left(I_{4c}\right)\;-\;B\left(I_{3b}\right) \end{array}$$

**Figure 6:**
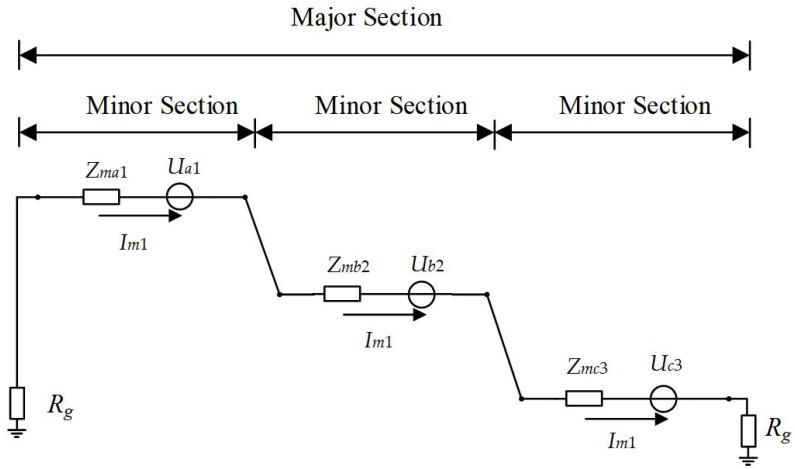
An equivalent circuit diagram of the sheath current.

Table 3 shows that when a single-phase short-circuit occurs, the phase difference of the fault cable segment is significantly higher than those non-fault cable segments, as the phase differences of the non-fault segments are very small. In fact, they are less than ±2°, as shown in the performed simulation. Consequently, the fault segment can be identified, based on the phase difference of the sheath currents at either end of the segment. It is to be noted that the results given in Table 3 are not exactly 0 or 180°. To explain the determining factors, an equivalent circuit diagram is shown in Figure 6.

In Figure 6, *I*_*m*1_ is denoted as the sheath current in a closed sheath circuit. *U_a_*_1_, *U_b_*_2_, *U_c_*_3_ are the equivalent voltage sources due to electromagnetic induction in the circuit. *Z*_*ma*1_, *Z*_*mb*2_, *Z*_*mc*3_ are equivalent impedances of segments A1, B2, and C3 respectively. *R_g_* is grounding resistance. *Z*_*ma*1_, *Z*_*mb*2_, *Z*_*mc*3_ include both the inductive reactance and the sheath resistance of each of the cable segments in the loop, while *R_g_* stands for the grounding resistance. The value of *R_g_* has an influence on both the magnitude and the phase angle of the sheath current Im1. When no fault occurs in the circuit, *I*m1 can be represented, as given in Equation (7).

(7) $$I_{m1}=\frac{U_{a1}+U_{b2}+U_{c3}}{Z_{ma1}+Z_{mb2}+Z_{mc3}+2R_{g}}$$

**Figure 7:**
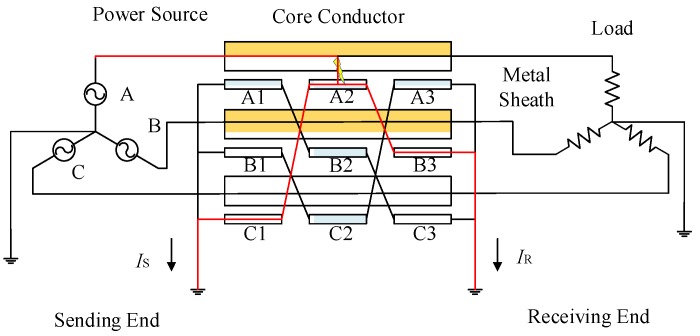
The short-circuit fault between the core conductor and the metal sheath.

Assuming that a single-phase short circuit fault occurs in segment A2, as shown in Figure 7, the current flowing in the core conductor from the power source to the fault point is the fault current, while the current flowing from the fault point to the load is nearly 0 A. The fault current flowing in the core conductor from the power source to the fault point induces much higher voltages in the metal sheath of cable segment A1 and part of A2, while the induced voltages in A3 and the other part of A2 is nearly 0 V. Meanwhile, there are higher induced sheath voltages at segments C1, A2, and B3. Due to the variation among the induced voltages, the sheath currents differ at every sensor position in the metal sheath circuit. For further illustration of the phase difference of the currents at either end of the fault cable segment, the circuit law is given in Equation (8) and the phase angle of *I* is shown in Equation (9), where *I* represents the current phasor, *U* the voltage phasor, *R* the resistance, *X* the reactance, *j* the imaginary unit, *θ* the phase angle of *I*, and *θ_U_* stands for the phase angle of *U*.

(8) $$I=\frac{U}{R+jX}$$

(9) $$\theta =\arctan\left(-\frac{X}{R}\right)+\theta_{U}$$

According to Kirchhoff’s circuit law, the sheath current flowing from the fault point to the sending end *I_S_* is shown in Equation (10). The sheath current flowing from the fault point to the receiving end *I_R_* is shown in Equation (11). Their phase angles are shown in Equations (12) and (13), where *U_f_* is the voltage of the fault point; *U_IS_* is the equivalent induction voltage of the sending end in the equivalent circuit; *U_IR_* is the equivalent induction voltage of the receiving end; *r_0_* is the equivalent sheath resistance per unit length; *x_0_* is the equivalent sheath reactance per unit length; *L_S_* is the length between the fault point and the sending end; *L_R_* is the length between fault point and the receiving end; *I_f_* is the fault current flowing through the core conductor; *I_nb_* represents the total currents in the other cable circuits laid in the same cable trench; *θ_S_* is the phase angle of *I_S_*; *θ_R_* is the phase angle of *I_R_*; *θ_US_* is the phase angle of (*U_f_* + *U_IS_*); *θ_UR_* is the phase angle of (*U_f_* + *U_IR_*).

(10) $$I_{S}=\frac{U_{f}+U_{IS}}{R_{g}+r_{0}L_{S}+jx_{0}L_{S}}$$

(11) $$I_{R}=\frac{U_{f}+U_{IR}}{R_{g}+r_{0}L_{R}+jx_{0}L_{R}}$$

(12) $$\theta_{S}=\arctan\left(-\frac{x_{0}L_{S}}{R_{g}+r_{0}L_{S}}\right)+\theta_{US}$$

(13) $$\theta_{R}=\arctan\left(-\frac{x_{0}L_{R}}{R_{g}+r_{0}L_{R}}\right)+\theta_{UR}$$

The phase difference of the current flow in the two directions (*I_S_* and *I_R_*) in the fault cable segment cannot be represented by a phasor expression as in Equation (9). The phase angle difference between *I_S_* and *I_R_* is approximately the difference between the *P*(segment) and 180°. As Equations (12) and (13) show, the difference between *θ_S_* and *θ_R_* mainly depends on the difference between *θ_US_* and *θ_UR_* when *R_g_* is very small. The difference between *θ_US_* and *θ_UR_* depends on the difference between *U_IS_* and *U_IR_*.

**Figure 8:**
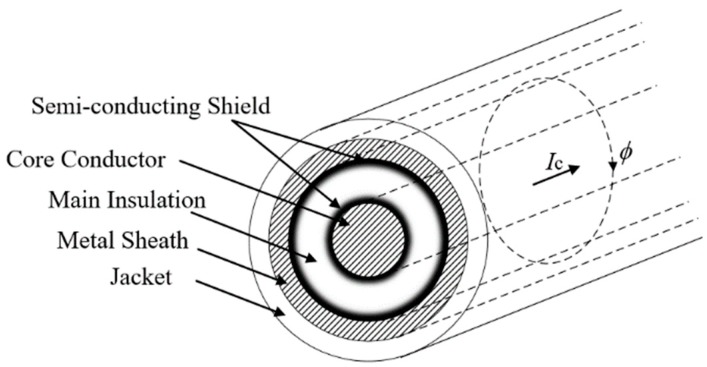
The core conductor current with its magnetic flux.

As shown in Figure 8, a sheath voltage is induced by the cross-linked magnetic flux *Φ* when a current *I*_c_ exists in the core conductor. Assuming that *r*_1_ is the core radius, *r*_2_ is the outer radius of main insulation; *r*_3_ is the outer radius of the metal sheath; *r*_4_ is the outer radius of jacket. The cross-linked magnetic flux of unit length *Φ* can be expressed in Equation (14). The induced voltage *e*_0_ on the metal sheath of unit length *e*_0_ can be expressed in Equation (15).

(14) $$\Phi =\int_{r_{2}}^{r_{3}}\frac{\mu_{0}I_{c}}{2\pi r}dr=\frac{\mu_{0}I_{c}}{2\pi }\ln\frac{r_{3}}{r_{2}}$$

(15) $$e_{0}=-\frac{d\Phi }{dt}$$

Let *i_c_*(*t*) = *I_m_* sin(*ωt* + *θ*), the complex form of *i_c_*(*t*) is denoted using *I*_c_. Where *I_m_* is the maximum of *i_c_*(*t*); *ω* is the angular frequency of *i_c_*(*t*), and *ω* = 2π*f*; *f* is the frequency of *i_c_*(*t*); *t* is the time variable, and *θ* is the time constant. The complex variable of *e*_0_ can be expressed as in Equation (16).

(16) $$E_{0}=-j\mu_{0}f\ln\frac{r_{3}}{r_{2}}\cdot I_{c}$$

The magnetic field can be changed by currents flowing through other cables within the same cable trench. However, the change is not remarkable due to the greater physical distance, and therefore the mutual coupling between adjacent cables is insignificant. Assuming that the change can be neglected, *U_IR_* ≅ 0, *U_IS_* can be expressed as Equation (17):

(17) $$U_{IS}\approx -j\mu_{0}f\ln\frac{r_{3}}{r_{2}}\cdot L_{S}\cdot I_{f}$$

As Equation (17) shows, the greater the value of (*L_S_*·*I_f_*), the greater is *U_IS_*. Meanwhile the phase difference becomes greater. *I_f_* is the main determining factor of the difference between *U_IS_* and *U_IR_*. *I_f_* is dependent on several factors, such as the grounding resistance, the fault position, the line voltage, and so on.

**Figure 9:**
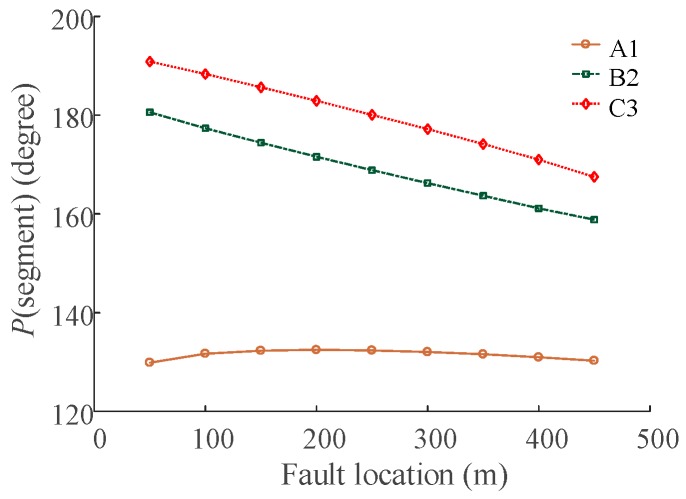
The relationship between *P*(segment) and the fault location.

**Figure 10:**
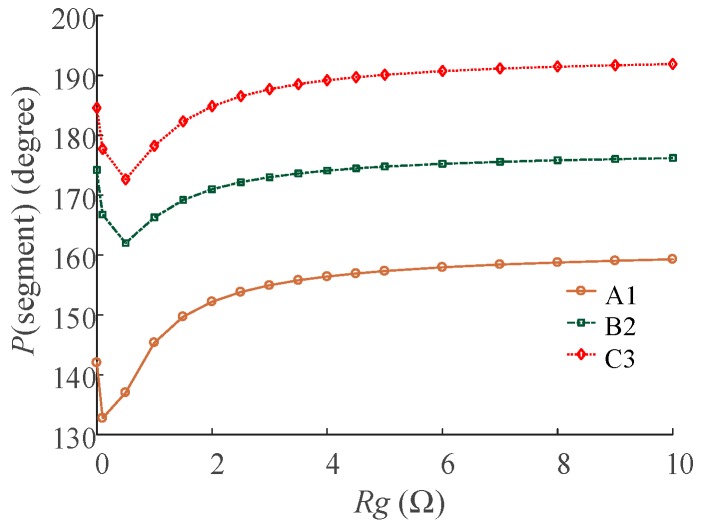
The relationship between *P*(segment) and *R_g._*

Further simulations were carried out to study the relation of *P*(segment), *R_g_*, and the fault position. First, the load was set as 40 MW. The grounding resistance *R_g_* was set as 0.1 Ω. The location of the fault was allowed to change in steps of 50 m, e.g., 50~450 m, from the sending end along each minor section. The results of *P*(segment) are shown in Figure 9 (segment ∈ {“A1”, “B2”, “C3”}). Second, the load was set to 40 MW. The location of fault point was set to 300 m from the sending end of each segment. The grounding resistance *R_g_* was allowed to change between 0~10 Ω. The results of *P*(segment) are shown in Figure 10 (segment ∈ {“A1”, “B2”, “C3”}).

The relationship between *P*(segment) and fault location is shown in Figure 9, and the relationship between *P*(segment) and *R_g_* is given in Figure 10. The *P*(segment) is a function of the grounding resistance, the load and the fault location, which means that *P*(segment) can be the criteria for fault location if the grounding resistance and the load can be obtained.

In this case, the fault segment can be identified without the need for accurate line and system parameters if the following two conditions are met:

(1) *P*(segment) ∈ [90°, 270°], segment ∈ Q, Q = {“A1”,“B1”,“C1”,“A2”,“B2”,“C2”,“A3”,“B3”,“C3”};
(2) *P*(segment’) ∈ [−10°, 10°], segment’∈ {x| x ∈ Q and x ≠ segment}.

The cable segment satisfying condition 1 above can be identified as the one with a fault. It is to be noted that if all the results of *P*(segment) meets condition 2 and none satisfies condition 1, the fault is outside the monitored cable section, or the fault is external.

**Figure 11:**
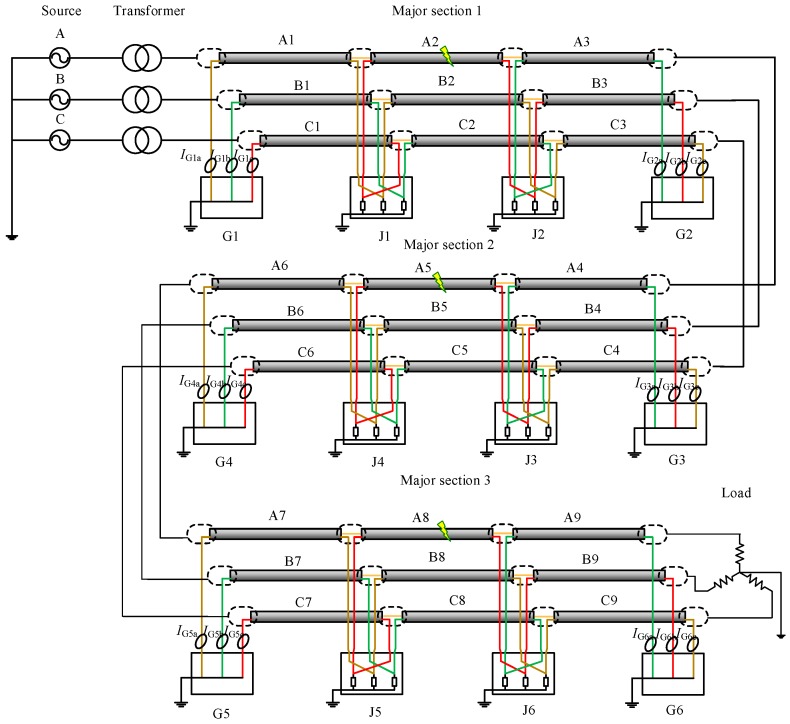
The schematic diagram of the power system with three major cable sections.

To further investigate the recognition of the external faults, a simple power system model with three major cable sections was simulated. The fundamental simulation parameters were the same as the simulated power network shown in Figure 4. Three rounds of simulation were carried out with the fault location set in cable segment A2, A5, and A8, respectively, as shown in Figure 11. Note that only the current sensors installed at G1, G2, G3, G4, G5, and G6 were of significance for recognition of external faults, and they are shown in the diagram. As there are three major sections, the first step is to locate the major section with the short-circuit fault. The phase difference of the sheath currents at two ends of each major section is given in Equation (18), where S1A represents the cable sheath loop A1–B2–C3; S1B represents the cable sheath loop B1–C2–A3, S1C represents the cable sheath loop C1–A2–B3; S2A represents the cable sheath loop A4–B5-C6; S2B represents the cable sheath loop B4–C5–A6; S2C represents the cable sheath loop C4–A5–B6; S3A represents cable sheath loop A7–B8–C9; S3B represents cable sheath loop B7–C8–A9, and S3C represents cable sheath loop C7–A8–B9. The simulation results are shown in Table 4.

(18) $$\begin{array}{l} P\left(S1A\right)=B\left(I_{G2c}\right)\;-\;B\left(I_{G1a}\right)\\ P\left(S1B\right)=B\left(I_{G2a}\right)\;-\;B\left(I_{G1b}\right)\\ P\left(S1C\right)=B\left(I_{G2b}\right)\;-\;B\left(I_{G1c}\right)\\ P\left(S2A\right)=B\left(I_{G4c}\right)\;-\;B\left(I_{G3a}\right)\\ P\left(S2B\right)=B\left(I_{G4a}\right)\;-\;B\left(I_{G3b}\right)\\ P\left(S2C\right)=B\left(I_{G4b}\right)\;-\;B\left(I_{G3c}\right)\\ P\left(S3A\right)=B\left(I_{G6c}\right)\;-\;B\left(I_{G5a}\right)\\ P\left(S3B\right)=B\left(I_{G6a}\right)\;-\;B\left(I_{G5b}\right)\\ P\left(S3C\right)=B\left(I_{G6b}\right)\;-\;B\left(I_{G5c}\right) \end{array}$$

As can be seen from Table 4 for the fault in cable section A2/section 1, which is external to the major sections 2 and 3, *P*(S2A), *P*(S2B), *P*(S2C), *P*(S3A), *P*(S3B), *P*(S3C) meet condition 2. The same conclusion can be drawn from the cases where the fault happens in the cable sections A5 and A8. These suggest that the external fault can be identified if all the results of *P*(segment) meet condition 2 and none satisfy condition 1.

### 4.3. Modified EMTR for Fault Point Location

After the fault segment is located, the next step is to locate the specific fault position in the cable segment where the fault happened. According to the EMTR [13,14,15,16], the fault position is where the greatest energy concentration appears. The location can be determined by a series of simulations of the back-injected time-reversed fault transients. The original EMTR method had the recorded signal reversed in the time-domain, before the energy of the signal for each a priori location (or “guessed fault location”) are calculated [16]. However, the location procedure could be simplified by analyzing the energy of the recorded signal in frequency–domain (without calculating a priori locations). The amount of a priori locations determined the computation for the original EMTR method, because the energy of the time-domain signal corresponds to each a priori location. While the energy of the frequency–domain signal could be calculated only once as a function with the independent variable of the “guessed fault location”. In addition, with the modification, the method can be applied to situations where only sheath currents are available.

The frequency–domain expressions of electromagnetic transients generated by the fault were established in [16] and presented in Equation (19), where *ρ*_1_ represents the reflection coefficient at the line terminal *x* = 0; *γ* represents the line propagation constant; *x_f_* represents the fault position; *I_A_*_1_ represents the current observed at line the terminal *x* = 0 in frequency domain; * represents the complex conjugate; and *I_f_*_1_ represents an equivalent current source at *x* = *x_f_*.

(19) $$I_{f1}\left(x_{f},\omega \right)=\frac{\left(1+\rho_{1}\right)e^{-\gamma x_{f}}}{1+\rho_{1}e^{-2\gamma x_{f}}}I_{A1}^{*}\left(\omega \right)$$

As *ρ*_1_ and *γ* are constants for a line with a given network topology and line parameters, *I_A_*_1_ can be obtained at the monitoring position (*x* = 0). Thus, *I_f_*_1_ is a function with an independent variable *x_f_*; the point where there is a maximum *I_f_*_1_ is the fault point.

**Figure 12:**
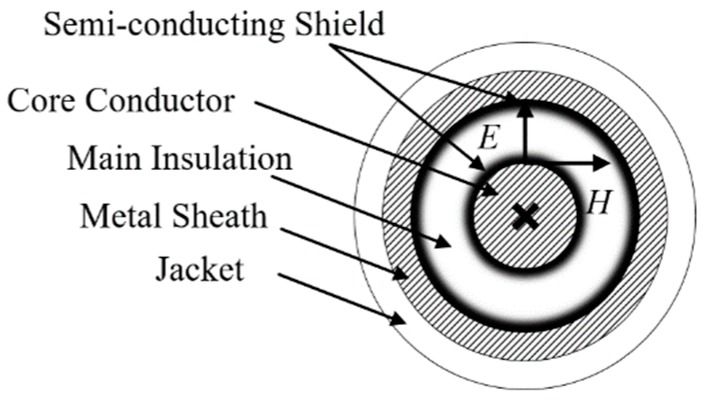
The electric and magnetic field directions for a typical HV cable.

For a typical HV cable structure, the electric and magnetic field directions (***E*** and ***H***) are presented in Figure 12. The energy propagates along the cable axis in the main insulation. As the traveling waves (fault transients) are essentially flows of energies, there is no difference between monitoring the waveform of traveling waves by setting the monitoring equipment at the core conductor or at the metal sheath.

**Figure 13:**
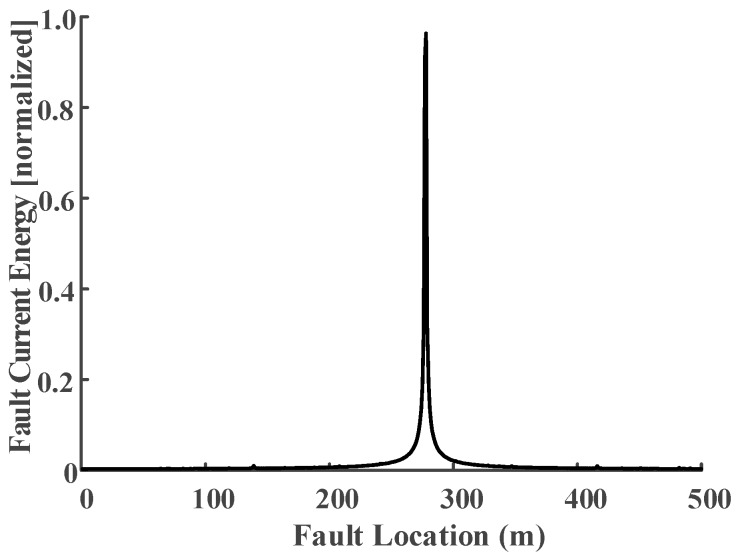
Normalized energy of the sheath current signal as a function of *x_f_*. The real fault location is *x_f_* = 300 m, the largest energy concentration is *x_f_* = 278 m.

The same simulation results (raw data of *I*_1*a*_) shown in Figure 5 can be used as the input to illustrate the EMTR procedure. The difference in the utilization of the monitored sheath currents is the requirement of higher sampling rate. The power–frequency of the sheath currents are needed for fault segment location, while the fault transients are used for fault point location. The full frequency domain fault transient is the *I_A_*_1_ in (19); thus, the function with the independent variable *x_f_* (0 ≤ *x_f_* ≤ 500) can be shown in Figure 13.

The result in Figure 13 shows the normalized energy of the sheath current *I*_1*a*_ within a frequency spectrum from dc to 5 MHz (sampling rate: 10 MHz), and it is also the function of *I*_1*a*_ versus the independent variable *x_f_*.

**Figure 14:**
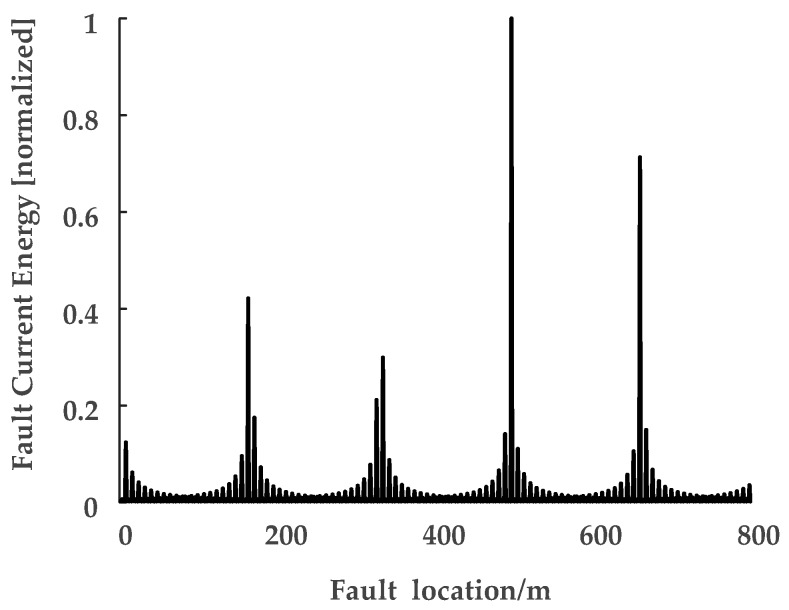
Normalized energy of the sheath current signal as a function of *x_f_*. The real fault location is *x_f_* = 500 m, the largest energy concentration is *x_f_* = 497 m.

Clearly, there is a location error between the real fault position and the largest concentration point, though it is still within engineering tolerance [25]. Simulations for faults occurring in other cable segments generated similar results. Further simulations have been carried out for an 800-long cable segment, where the sampling rate was set as 100 MHz. The result of the fault current energy with the independent variable *x_f_* (0 ≤ *x_f_* ≤ 800) is shown in Figure 14.

**Figure 15:**
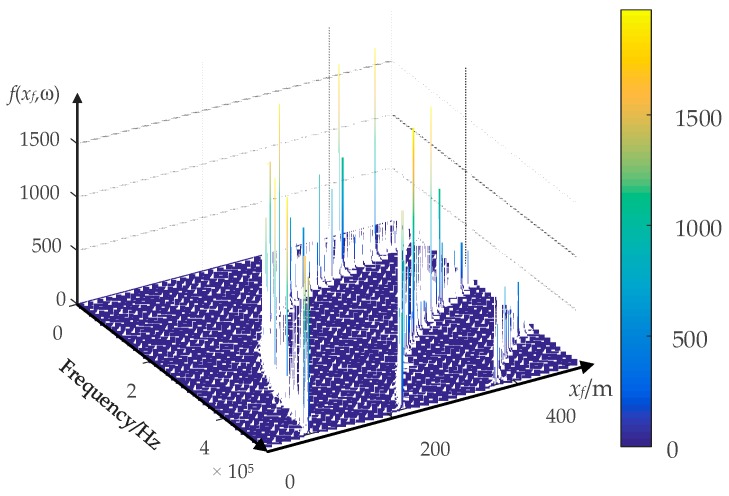
The electromagnetic transients transfer function *f*(*xf*,ω) under the sampling rate of 1 MHz.

**Figure 16:**
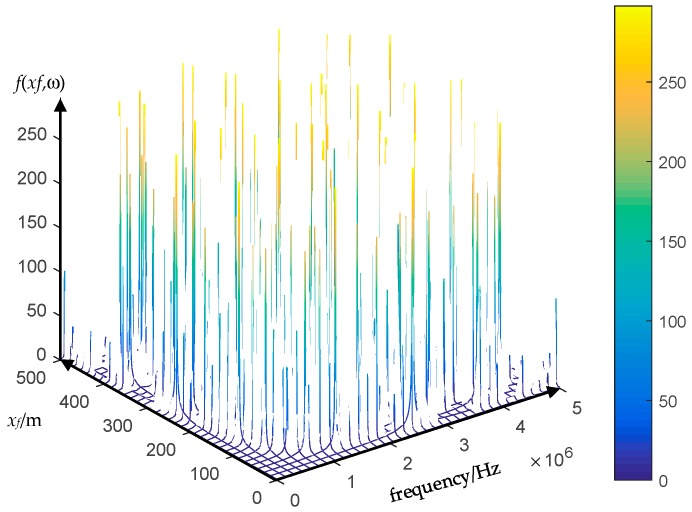
The electromagnetic transients transfer function *f*(*xf*,ω) under the sampling rate of 10 MHz.

**Figure 17:**
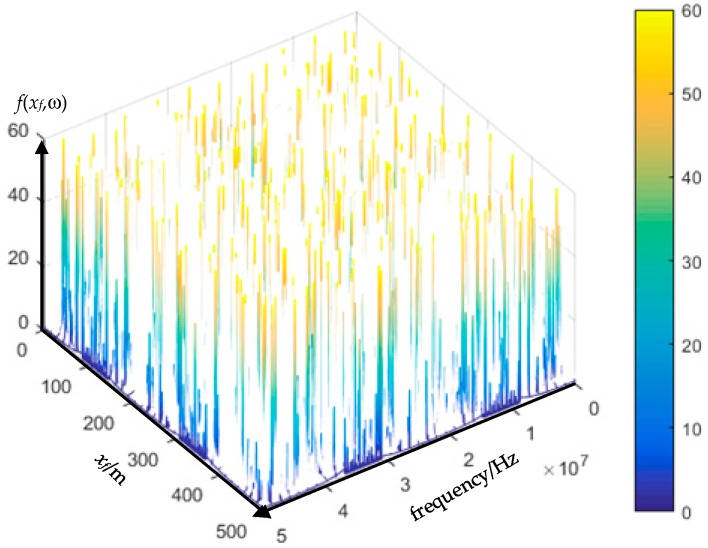
The electromagnetic transients transfer function *f*(*x_f_*,ω) under the sampling rate of 100 MHz.

The location accuracy of the result shown in Figure 13 is better than the result shown in Figure 12. Theoretically, the fault position is the largest energy concentration position. However, the fault location accuracy depends on the accuracy of the electromagnetic transients transfer function (Equation (19)). The sampling error can cause inaccuracies in the FFT spectral energy analysis, which eventually leads to inaccuracy of the electromagnetic transients transfer function. The figures of the electromagnetic transients transfer function under different sampling rates are presented in Figure 15, Figure 16 and Figure 17.

The transfer function *f*(*x_f_*,ω) of electromagnetic transients differs with the sampling rate as presented in Figure 15, Figure 16 and Figure 17, and it is not a “smooth” function. It is a grass-like shape, or it becomes denser as the sampling rate increases. The higher sampling rate, the closer the results are to the reality. Therefore, the simulation results suggest that the sampling rate should be 10 MHz or higher.

### 4.4. Case Study—An External Fault detected by the Online Cable Fault Location System

**Figure 18:**
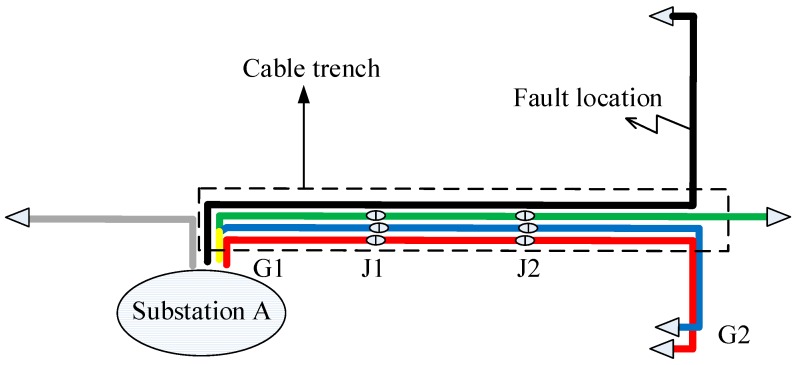
The cable passage of the case study.

**Figure 19:**
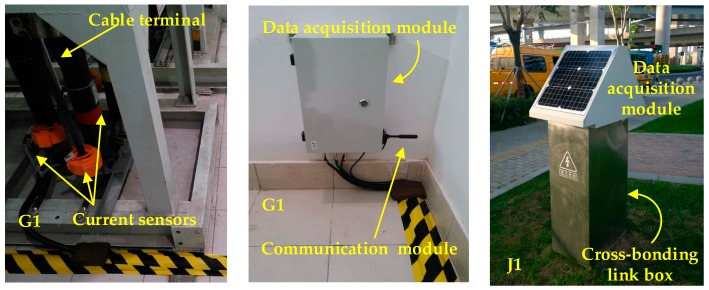
The on-site installation pictures of the case study (the first two pictures were taken in an indoor substation, the last picture was taken outdoors).

In order to obtain the fault data in real HV cable systems and to confirm the effectiveness of the online fault location system, a 110 kV cable circuit with a major section was chosen to be implemented with the proposed online fault location system. The connections of the cable section where the sensors and data acquisition systems were installed is shown in Figure 1. The 110 kV HV cable circuit is in the city of Suzhou, China. The cable passage is shown in Figure 18, where G1, J1, J2, G2 are the four current sensor installation positions, as described in Section 2 of the paper, and the on-site installation is shown in Figure 19. The first two pictures in Figure 19 were taken in the indoor substation A (G1). The data acquisition module and the communication module was directly installed indoors using the power supply of the substation. The cross-link boxes are above the ground in Suzhou, as shown in the last picture of Figure 19. The data acquisition module and the communication module was installed in a custom cabinet, and a couple of solar panels were used for power supply.

**Figure 20:**
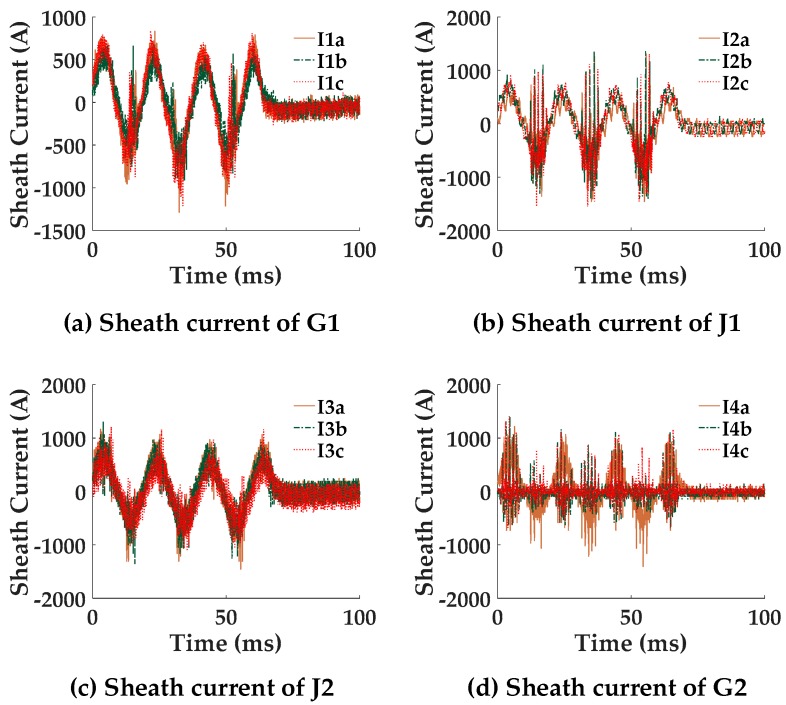
The sheath currents at each of the sensor locations.

As shown in Figure 18, the same substation also feeds other cable circuits, some in the same cable trench, including two 35 kV and three 110 kV cable circuits, which were started from substation A. The red, blue, and green lines (the bottom three circuits) in the diagram represented the three 110 kV cable circuits, whilst the black and gray lines represented the 35 kV circuits. Four cable circuits share the same cable trench, of which the 3-phase circuit in the bottom and marked in red was the one equipped with an online monitoring system. On 8 December 2016, a fault occurred in the black-colored 35 kV cable circuit. Before the fault was cleared, all four sets of data acquisition equipment were triggered due to the sheath currents in the HV cable exceeding the preset alarm level of 70 A. (The preset trigger current in the system designed by the authors is 70 A. This low level of trigger current allowed high-resistance faults to be detected. In fact, the equipment could be triggered even if the fault resistance is as high as 907 Ω in the 110 kV line. It is true that the minor sections hardly have equal lengths in practice, which can cause relatively high sheath currents under normal operation. However, under normal operation conditions, this circulating current rarely reaches a level of 30 A, due to unequal lengths among the minor sections. In case of the extreme cases where cable sheath circulating under normal conditions can reach a level of 70 A, the trigger current can be raised. The level of the sheath current can be readily evaluated in advance using the model given in [16]) The sheath currents recorded by the proposed fault location system are shown in Figure 20.

**Figure 21:**
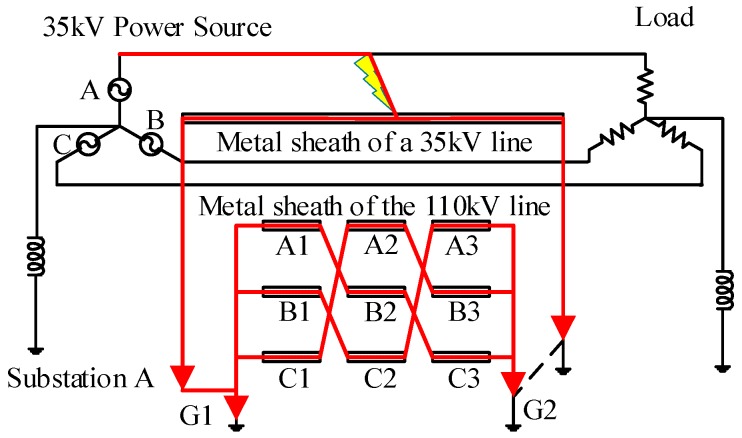
The equivalent circuit of fault current.

Although the signal noise level was high as Figure 20 shows, the fundamental phase difference of each segment *P*(segment) is almost 0, which means the fault did not originate from any of the sections equipped with the online monitoring equipment. The specific results of each phase difference *P*(segment) are all presented in Table 5. They all met condition 2 of the criteria for fault segment location, which suggests that the fault was external to the cable section being monitored. For further research, the equivalent circuit representing the circuit connections at the time that the fault happened is shown in Figure 21.

When the fault occurred in the 35 kV cable circuit, which was connected to the same substation as the HV circuit being monitored, the fault current flowed into ground through the metal sheath of the 35 kV cable system. Meanwhile, part of the fault current flowed into ground directly at substation A (G1). The fault current also flowed through the metal sheath of the 110 kV circuit as it formed part of the fault current path. This resulted in the excessive level of sheath current that triggered the fault location equipment.

The external fault was caused by a short-circuit fault outside the cable circuit in which the monitoring system was installed. However, the metal sheath of the fault cable circuit shared the same grounding network with the cable equipped with the online monitoring system. The detection and correct classification of the external fault by the monitoring system helped to prove the effectiveness of the monitoring system.

## 5. Conclusions

This paper proposes a novel approach for fault location in a cross-bonded cable major section. Fault segment can be identified via the acquisition and analysis of cable sheath currents. The sheath current direction can be analyzed via their power–frequency component. The difference in the power-frequency phase of the sheath currents at either end of each minor segment can be used for fault segment location. Because the current would flow in opposite directions for the cable segment where there is a fault, whilst for other segments without fault, sheath currents would flow in the same direction. The fault location method and the online condition monitoring system have been proven to be effective in the case study shown in the paper.

The present paper has focused on the most popular cross bonding scheme, while there are other cross-bonding schemes such as single-point bonding, multiple single-point bonding, impedance bonding, sectionalized cross bonding, and continuous cross bonding [18,22]. The criteria for fault segment location of different cross bonding schemes, as well as more than one major cable section in a circuit, will be investigated in the future. However, the fundamental theory of the location method is the same.

The paper also explored the effectiveness of a modified EMTR in the HV cable system. The transient component of the sheath current can be used to locate the fault using numerical simulations. The energy consumption of the travelling wave during propagation and the sampling error can result in a location error. The sampling rate should be 10 MHz or higher to ensure that the location error is within engineering tolerance.

## Figures and Tables

**Table 1:** Parameters of the cross-sectional Structure of the cable.

	Structure	Outer Radius/mm
1	Core conductor (copper)	17.0
2	Inner semi-conductor (nylon belt)	18.4
3	Main insulation (ultra-clean XLPE)	34.4
4	Outer semi-conductor (super-smooth semi-conductive shielding material)	35.4
5	Water-blocking layer (semi-conductor)	39.4
6	Metal sheath (aluminum)	43.9
7	Jacket (PVC)	48.6

**Table 2:** The current magnitude at each sensor position under different fault conditions.

Fault Segment	A1	B1	C1	A2	B2	C2	A3	B3	C3
*Ma*(*I*_1*a*_) *	6808	148	150	2913	4720	719	2078	2140	2655
*Ma*(*I*_1*b*_) *	153	6808	147	1025	1515	4885	2658	2143	2072
*Ma*(*I*_1*c*_) *	149	152	6805	6314	856	1751	2076	2721	2076
*Ma*(*I*_2*a*_) *	1134	151	146	2912	4723	716	2077	2142	2652
*Ma*(*I*_2*b*_) *	150	1135	149	1022	1513	4888	2654	2142	2075
*Ma*(*I*_2*c*_) *	152	149	1138	6315	852	1750	2078	2717	2075
*Ma*(*I*_3*a*_) *	152	151	1141	4184	854	1747	2077	2719	2072
*Ma*(*I*_3*b*_) *	1137	151	149	2909	2842	715	2074	2142	2654
*Ma*(*I*_3*c*_) *	152	1138	149	1022	1510	2746	2656	2139	2074
*Ma*(*I*_4*a*_) *	152	1139	145	1022	1513	2750	4808	2142	2071
*Ma*(*I*_4*b*_) *	148	151	1141	4188	854	1750	2074	4759	2075
*Ma*(*I*_4*c*_) *	1138	147	149	2911	2846	715	2077	2138	4813

* *Ma*(*I*) is magnitude of the fundamental signal of *I*. the unit is A.

**Table 3:** Phase difference of the currents at the two ends of minor cable segments.

Fault Segment	A1	B1	C1	A2	B2	C2	A3	B3	C3
*P*(A1) *	132	0.21	1.17	0.03	0.11	1.14	0.01	0.2	−0.18
*P*(B1) *	1.18	132	0.23	1.13	0.03	0.11	−0.18	0.01	0.2
*P*(C1) *	0.24	1.18	132	0.11	1.15	0.03	0.22	−0.18	0.01
*P*(A2) *	−0.04	−0.11	0.03	175	0.03	0.21	0.00	0.01	0.05
*P*(B2) *	0.04	−0.10	0.00	0.22	166	0.06	0.08	0.00	0.01
*P*(C2) *	−0.22	0.04	−0.08	0.06	0.28	158	0.01	0.06	0.00
*P*(A3) *	−0.08	0.01	0.07	−0.08	0.03	0.00	177	0.03	0.07
*P*(B3) *	0.07	−0.08	0.01	0.07	−0.08	0.03	0.07	176	0.03
*P*(C3) *	0.01	0.07	−0.08	0.03	0.07	−0.08	0.03	0.07	177

* The unit of *P*(segment) and *B*(*I*) is degrees.

**Table 4:** Phase difference of the currents at the two ends of major cable sections.

Fault Section	A2	A5	A8
*P*(S1A) *	0.05	0.03	0.03
*P*(S1B) *	0.15	0.03	0.03
*P*(S1C) *	175.52	0.03	0.03
*P*(S2A) *	−3.07	0.06	0.04
*P*(S2B) *	−3.02	0.17	0.04
*P*(S2C) *	−2.86	175.61	0.04
*P*(S3A) *	−3.11	−3.11	0.07
*P*(S3B) *	−3.07	−3.07	0.19
*P*(S3C) *	−2.90	−2.91	175.68

* The unit of *P*(section) is °.

**Table 5:** Phase difference of the currents at two end of minor cable sections.

Minor Section	A1	B1	C1	A2	B2	C2	A3	B3	C3
*P*(segment) *	2.81	0.31	9.41	2.74	8.72	4.78	2.36	7.89	5.03

* The unit of *P*(segment) is °.

## References

[1] Sheng B., Zhou W., Yu J., Meng S., Zhou C., Hepburn D.M. (2014). On-line PD detection and localization in cross-bonded HV cable systems. IEEE Trans. Dielectr. Electr. Insul..

[2] Zhang X., Gockenbach E., Wasserberg V., Borsi H. (2007). Estimation of the lifetime of the electrical components in distribution networks. IEEE Trans. Power Deliv..

[3] Tang Z., Zhou C., Jiang W., Zhou W., Jing X., Yu J., Alkali B., Sheng B. (2014). Analysis of significant factors on cable failure using the cox proportional hazard model. IEEE Trans. Power Deliv..

[4] Dong X., Yuan Y., Gao Z., Zhou C., Wallace P., Alkali B., Sheng B., Zhou H. Analysis of cable failure modes and cable joint failure detection via sheath circulating current. Proceedings of the IEEE Electrical Insulation Conference (EIC).

[5] Eissa M.M. (2006). Ground distance relay compensation based on fault resistance calculation. IEEE Trans. Power Deliv..

[6] Xu Z.Y., Jiang S.J., Yang Q.X., Bi T.S. (2009). Ground distance relaying algorithm for high resistance fault. IET Gener. Transm. Distrib..

[7] Suonan J., Qi J. (2004). An accurate fault location algorithm for transmission line based on R – L, model parameter identification. Electr. Power Syst. Res..

[8] Novosel D., Hart D.G., Udren E., Garitty J. (1996). Unsynchronized two-terminal fault location estimation. IEEE Trans. Power Deliv..

[9] Girgis A.A., Fallon C.M. (1992). Fault location techniques for radial and loop transmission systems using digital fault recorded data. IEEE Trans. Power Deliv..

[10] Ji T., Sun T.J., Xu B.Y., Chen P., Xue Y.D. (2006). Study on fault location of distribution mixed feeders based on double terminal method of traveling waves. Proc. CSEE.

[11] Bawart M., Marzinotto M., Mazzanti G. (2016). Diagnosis and location of faults in submarine power cables. IEEE Electr. Insul. Mag..

[12] Dashti R., Salehizadeh S., Shaker H., Tahavori M. (2018). Fault Location in Double Circuit Medium Power Distribution Networks Using an Impedance-Based Method. Appl. Sci..

[13] Manesh H.M., Lugrin G., Razzaghi R., Romero C., Paolone M., Rachidi F. A new method to locate faults in power networks based on Electromagnetic Time Reversal. Proceedings of the 2012 IEEE 13th International Workshop on Signal Processing Advances in Wireless Communications (SPAWC).

[14] Lugrin G., Razzaghi R., Rachidi F., Paolone M. Electromagnetic time reversal applied to fault detection: The issue of losses. Proceedings of the 2015 IEEE International Symposium on Electromagnetic Compatibility (EMC).

[15] Razzaghi R., Lugrin G., Rachidi F., Paolone M. (2017). Assessment of the influence of losses on the performance of the electromagnetic time reversal fault location method. IEEE Trans. Power Deliv..

[16] Razzaghi R., Lugrin G., Manesh H., Romero C., Paolone M., Rachidi F. (2013). An efficient method based on the electromagnetic time reversal to locate faults in power networks. IEEE Trans. Power Deliv..

[17] Zhou C., Yang Y., Li M., Zhou W. An integrated cable condition diagnosis and fault localization system via sheath current monitoring. Proceedings of the 2016 International Conference on Condition Monitoring and Diagnosis (CMD).

[18] Li M., Zhou W., Wang C., Yao L., Su M., Huang X., Zhou C. A novel fault localization method based on monitoring of sheath current in a cross-bonded HV cable system. Proceedings of the 2017 IEEE Electrical Insulation Conference (EIC).

[19] Phanthurat S., Pruksanubal A. (2015). Sheath Voltages and Currents in 230kV Oil-Filled Underground Power Cables. Appl. Mech. Mater..

[20] Marzinotto M., Mazzanti G. (2015). The feasibility of cable sheath fault detection by monitoring sheath-to-ground currents at the ends of cross-bonding sections. IEEE Trans. Ind. Appl..

[21] Yang Y., Hepburn D.M., Zhou C., Jiang W., Yang B., Zhou W. On-line Monitoring and Trending Analysis of Dielectric Losses in Cross-bonded High Voltage Cable Systems. Proceedings of the 9th International Conference on Insulated Power Cables.

[22] Gustavsen B., Semlyen A. (1998). Combined phase and modal domain calculation of transmission line transients based on vector fitting. IEEE Trans. Power Deliv..

[23] Special Bonding of High Voltage Power Cables. https://e-cigre.org/share/publication/503/283-special-bonding-of-high-voltage-power-cables.

[24] Wedepohl L.M., Nguyen H.V., Irwin G.D. (1996). Frequency-dependent transformation matrices for untransposed transmission lines using newton-raphson method. IEEE Trans. Power Syst..

[25] Barry B.A., Morris M.D. (1978). Errors in Practical Measurement in Science, Engineering, and Technology.

